# Exploring the Impact of Restricted Partners’ Visiting Policies on Non-Infected Mothers’ Mental Health and Breastfeeding Rates during the COVID-19 Pandemic

**DOI:** 10.3390/ijerph18126347

**Published:** 2021-06-11

**Authors:** Daniela Morniroli, Alessandra Consales, Lorenzo Colombo, Elena Nicoletta Bezze, Lidia Zanotta, Laura Plevani, Monica Fumagalli, Fabio Mosca, Maria Lorella Giannì

**Affiliations:** 1 Fondazione IRCCS Ca’ Granda Ospedale Maggiore Policlinico, Neonatal Intensive Care Unit, 20122 Milan, Italy; daniela.morniroli@unimi.it (D.M.); lorenzo.colombo@policlinico.mi.it (L.C.); elena.bezze@policlinico.mi.it (E.N.B.); lidia.zanotta@policlinico.mi.it (L.Z.); laura.plevani@policlinico.mi.it (L.P.); monica.fumagalli@unimi.it (M.F.); fabio.mosca@unimi.it (F.M.); maria.gianni@unimi.it (M.L.G.); 2 Department of Clinical Sciences and Community Health, University of Milan, 20122 Milan, Italy

**Keywords:** breastfeeding, COVID-19, maternal anxiety, hospital policies

## Abstract

Changes in perinatal care occurring during the coronavirus disease 2019 (COVID-19) pandemic may negatively affect mothers’ mental health and breastfeeding. This study, performed between April and May 2020, aimed to investigate the effect of restricted partners’ visiting policies on non-infected mother’s anxiety symptoms, the perceived postpartum support, and the breastfeeding outcomes at discharge. A cross-sectional study was conducted in a neonatal tertiary referral center in northern Italy during Italy’s lockdown. We enrolled mothers with a negative nasopharyngeal swab for severe acute respiratory syndrome coronavirus 2 (SARS-CoV-2), adequate oral and written comprehension of the Italian language, and absence of underlying maternal or neonatal clinical conditions. Maternal anxiety levels were assessed through the State-Trait Anxiety Inventory-Form Y (STAI-Y). Maternal perception of staff’s support was evaluated by the Nurse Parent Support Tool (NPST). A STATE-A (concurrent emotional state after a specific situation) score ≥ 40 was considered indicative of clinically significant symptoms of anxiety. A total of 109 mothers completed the study. Mean STATE-A score was ≥40 in 42% of mothers, and median NPST score was 4.23. Mothers separated from their partner had a mean STATE-A score ≥ 40 in a higher percentage of cases than those who were not (51% vs. 30%, *p* = 0.03) and a lower perception of caregiver support. A NPST score ≤4.23, partner ‘s absence during the hospital stay and primiparity were independently associated with a STATE-A score ≥ 40. Breastfeeding rates at discharge were not influenced by maternal anxiety levels and partner’s restricted policies. Instead, they were influenced by mode of delivery, a well-known risk factor, and pre-pandemic intention to breastfeed. Our study demonstrates the positive impact of a partner’s presence on maternal mental health and perception of caregiver support.

## 1. Introduction

In January 2020, the World Health Organization declared a Public Health Emergency of International Concern as the coronavirus disease 2019 (COVID-19) was rapidly spreading across countries [1]. Since then, public health systems worldwide have been facing a twin challenge: at first, the main issue was how to address the ongoing tide of critical patients and contain the infection; secondarily, but no less critical, healthcare systems directed their efforts towards the management of essential hospital activities.

As general lockdown and social distancing were proclaimed [2], studies conducted worldwide began to show the impact of these practices on mental health, reporting an increased rate of anxiety symptoms and high-stress levels [3].

Pregnant women and newly mothers’ well-known increased risk for anxiety and depression exposes them to an even higher risk of psychological distress during a pandemic [4,5]. This is of importance as prenatal and postnatal maternal anxiety [6,7] has been reported to negatively affect breastfeeding which, in turn, contributes to the maintenance of mothers’ good physical and mental health [8,9].

In-hospital birth and postnatal care are essential services. Their handling in this peculiar situation put maternity wards under unusual pressure, leading to the change of their organizational and operational policies to protect women, newborns, and healthcare professionals in a novel setting that hardly offered any evidence-based best practice recommendation. Hospital policies regarding partners’ presence and external visits have, therefore, been revised [10], often leading to the precautionary separation of new mothers and newborns from friends, family members, and, in most cases, newborns’ co-parent too, during the hospital stay. These changes in perinatal care may negatively affect mothers’ emotional state and breastfeeding despite its well-known health benefits for both the mother and the infant [11,12]. Global guidance on pregnancy and puerperium management has highlighted the importance of closely monitoring mothers’ mental health during hospital stay [10], and the promotion and protection of breastfeeding during the COVID-19 pandemic has been advocated [12].

The present study aimed to investigate the effect of partners’ restricted visiting policies on newly mother’s anxiety symptoms throughout hospital stay during the current pandemic, the perceived postpartum support, and the breastfeeding outcomes at discharge.

## 2. Materials and Methods

### 2.1. Study Design

We performed a cross-sectional study in our Centre’s postnatal Unit between April and May 2020, during Italy’s lockdown [13]. Our hospital is a neonatal tertiary referral centre, covering around 6000 pregnancies per year. It is located in Lombardy, Northern Italy, one of the Italian regions first and most severely affected by the current pandemic [14]. The study was approved by the Institutional Ethics Committee “Comitato Etico Area 2 Milano” (Approval ID: 249_2020bis), and written informed consent was obtained from all participants.

### 2.2. Study Population

Enrollment began on 7 April 2020 and was completed on 10 May 2020. Considering the ongoing pandemic situation and the rapid changing hospital policies, we did not set an a priori study size.

A registered nurse or a neonatologist not involved in the dyad’s (mother and newborn pair) care assessed all mothers admitted to the postnatal unit within 48 h of admission. Inclusion criteria were: adequate oral and written comprehension of the Italian language, absence of underlying maternal or neonatal clinical conditions potentially impeding breastfeeding, a negative nasopharyngeal swab for severe acute respiratory syndrome coronavirus 2 (SARS-CoV-2), and signed written informed consent. Exclusion criteria were: inadequate oral and written comprehension of the Italian language, underlying maternal or neonatal clinical conditions impeding breastfeeding, contraindications to breastfeeding (e.g., previous mastectomy, drugs incompatible with breastfeeding, chemotherapy), declared intention not to breastfeed upon admission to the neonatal ward, a positive nasopharyngeal swab for SARS-CoV-2, and refusal to participate in the study. 

We decided not to include mothers with a positive nasopharyngeal swab for SARS-CoV-2 as being tested positive for COVID-19 could have, per se, further exacerbated maternal anxiety.

Mothers were enrolled after birth within 48 h of admission to the postnatal ward and were requested to fill out the questionnaire created for the present study during the hospital stay before discharge. At discharge, a registered nurse or a neonatologist collected the paper-based questionnaires.

### 2.3. Newly Implemented Hospital Policies

#### 2.3.1. Nasopharyngeal Swabs

Consistent with our centre’s written protocol, every mother in labour underwent a nasopharyngeal swab. Our centre’s laboratory immediately processed samples obtained before 4 pm, and results came back in 6–8 h; samples obtained after 4 pm were processed the next day.

#### 2.3.2. Partners and Visits

Our postnatal Unit includes two types of rooms: Italian Public Healthcare System rooms (i.e., rooms paid for by the Italian Public Healthcare System-Servizio Sanitario Nazionale, SSN) and private rooms (i.e., paid for by the patients, either directly or through insurance). SSN rooms are double or triple rooms, whereas private rooms are single rooms. Health workers (gynaecologists, obstetricians, neonatologists, and nurses) who look after mothers and their newborns are the same, regardless of the type of room occupied. Likewise, breastfeeding promotion is offered to all dyads, following the Baby-Friendly Hospital Initiative principles and the World Health Organization/United Nations Children’s Fund 10 Steps to Successful Breastfeeding [15].

Since the beginning of the pandemic, visits to the postnatal Unit of our Center have been suspended, both for SSN and private patients. Partners’ daily and overnight presence has been allowed only in private rooms to guarantee social distancing and avoid gatherings in SSN rooms.

#### 2.3.3. Rooming-In and Breastfeeding

Rooming-in was allowed for mothers who tested negative for SARS-CoV-2. Following the Italian Society of Neonatology [16], asymptomatic or paucisymptomatic (with few clinical symptoms) mothers who tested positive for SARS-CoV-2 or were still awaiting nasopharyngeal swab results stayed in a dedicated area of the postnatal unit with their newborns. Conversely, rooming-in was not allowed for mothers who tested positive for SARS-CoV-2 or were still awaiting results, in need of respiratory support or supplemental oxygen, with a temperature higher than 38 °C, impaired vital signs or unable to take care of the baby. Breastfeeding was recommended to every dyad, regardless of maternal SARS-CoV-2 status, provided that the appropriate mother-newborn infection control measures were implemented. 

### 2.4. Instrument

The questionnaire used for the present study consisted of 4 subsections: 1. Sociodemographic information; 2. Breastfeeding; 3. Nurse Parent Support Tool (NPST); 4. State-Trait Anxiety Inventory-Form Y (STAI-Y). A neonatologist, an International Board Certified Lactation Consultant, a registered nurse, and a neonatology resident created subsections 1–2. The first subsection included multiple-choice questions on maternal age, marital status, level of education, antenatal class attendance, breastfeeding as a topic addressed during antenatal class, mode of delivery, parity, current neonatal mode of feeding (exclusive breastfeeding, complementary breastfeeding and exclusive formula feeding according to the World Health Organization definition). Subsection 2 consisted of four questions: two multiple-choice and two open-ended questions. The first two questions addressed mothers’ pre-pandemic (a priori) and current intentions regarding breastfeeding. The two open-ended questions asked to specify how many months the mother intended to breastfeed or why she had decided not to breastfeed, depending on the answers given to the previous two multiple-choice questions.

The NPST [17] is a 5-point Likert scale questionnaire used to assess parents’ perception of nursing support during their infant hospitalization. The 21 items included in the questionnaire can be divided into four categories: Informational Support (nine items), Emotional Support (three items), Appraisal/Parental Esteem Support (four items), and Caregiving Support (five items). Scores for each item range from 1 (“Almost never”) to 5 (“Almost always”); higher scores show greater perceived support provided by the nursing staff. For each NPST, a total mean score and a subtotal mean score for each category were calculated. The validity and reliability of the NPST Italian version has been assessed by Montirosso et al. [18].

The STAI-Y, for which a validated Italian version is available [19], is a self-assessment questionnaire commonly used to detect and evaluate anxiety both as a personality trait (TRAIT-A) and as a concurrent emotional state after a specific situation (STATE-A). The STAI-Y comprises 40 questions (20/20). All items are rated on a 4-point Likert scale (from “Almost Never” to “Almost Always,” or from “Not at all” to “Very much so”). Total scores for each part range from 20 to 80. Higher scores indicate more significant anxiety. A score ≥ 40 is considered indicative of clinically significant anxiety symptoms [20].

Mothers enrolled were offered the possibility to choose between a paper-based or online questionnaire. The online questionnaire was created by a neonatology resident using Google Forms (Google LLC, Mountain View, CA, USA), and a link to the online form was sent to mothers via email from a Google account specially created for the present study. The paper-based and the online questionnaire were otherwise identical. Anonymity was guaranteed through the use of an alphanumeric code each mother was given at enrollment. The questionnaire took approximately 30 min to be filled out. Answers to online questionnaires were automatically inserted in an Excel spreadsheet, whereas answers from paper-based questionnaires were manually inserted in the same Excel spreadsheet by a neonatology resident. All data analyzed for the present study were obtained from the questionnaire, except neonatal data, retrieved from neonatal computerized medical charts (Neocare, i & t Informatica e Tecnologia Srl, Trento, Italy).

Before enrollment began, we asked a group of 5 mothers to evaluate the questionnaire’s comprehensibility and whether the time required to complete it was excessive. The NPST and STAI-Y could not be modified. According to the mothers’ suggestions, we modified the two final questions from close to open-ended. Mothers also stated that the questionnaire did not require too much effort to be completed, and participation in the study was compatible with their newborn’s care.

### 2.5. Statistical Analysis

Data were analyzed from 11 May through 20 May 2020. All participants who completed the questionnaire were included in the analysis. Categorical variables were expressed as numbers (frequencies) and compared between groups using the χ^2^ test. Continuous variables were tested for normality using the Kolmogorov–Smirnov test and expressed as mean and standard deviation (SD) or median and interquartile range (IQR), depending on the normal or non-normal distribution of the variable, respectively. Continuous variables were subsequently compared between groups with the independent samples *t*-test or non-parametric tests, as appropriate.

Both the NPST and the STAI-Y showed good internal consistency (Cronbach’s α = 0.96 and 0.93, respectively).

Univariate binary logistic regression models were used to examine associations between STATE-A score (≥40 vs. <40) and variables of interest: TRAIT-A score (≥40 vs. <40), NPST score (≤4.23 vs. >4.23), maternal age (≤35 years vs. >35 years), parity (primiparous vs. multiparous), mode of delivery (caesarean section vs. vaginal delivery), marital status (single vs. in a stable relationship), maternal education (>13 vs. ≤13 years), type of room occupied (SSN room vs. private room). Univariate binary logistic regression models were further used to examine associations between exclusive breastfeeding at discharge (yes vs. no) and variables of interest: maternal age (≤35 vs. >35 years), parity (primiparous vs. multiparous), mode of delivery (caesarean section vs. vaginal delivery), STATE-A score, (≥40 vs. <40), TRAIT-A score (≥40 vs. <40), NPST score (≤4.23 vs. >4.23), ante-natal class attendance (yes vs. no), a priori choice to exclusively breastfeed (yes vs. no), marital status (single vs in a stable relationship), maternal education (>13 vs ≤13 years), type of room occupied (SSN room vs. private room). Variables significantly associated with the outcome were then fit in multivariable logistic regression models. For analysis purposes, NPST scores and maternal age were divided into two groups according to their median values.

Statistical significance was set at two-sided *p* < 0.05. Statistical analysis was performed with SPSS version 21 statistic software package (SPSS Inc., Chicago, IL, USA).

## 3. Results

**Figure 1:**
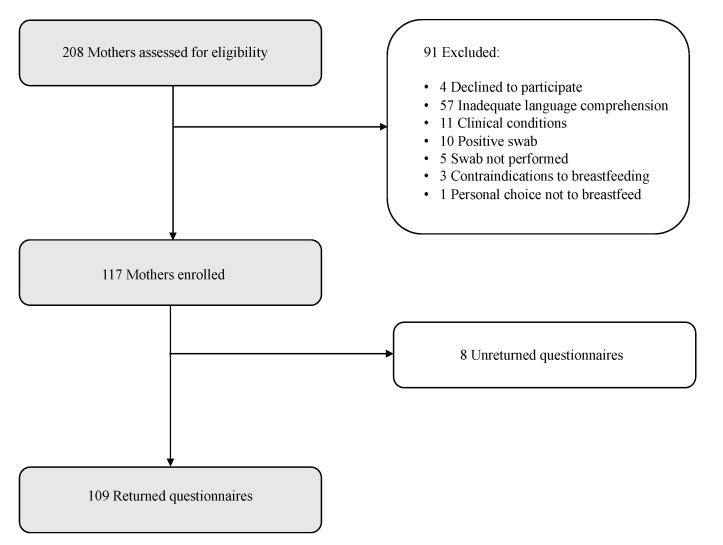
Patient flow through the present survey study.

Of 208 mothers assessed for eligibility, 117 were enrolled (Figure 1). Four mothers refused to participate in the study, 87 were excluded based on the exclusion criteria: 56 for inadequate comprehension of the Italian language, 3 for contraindications to breastfeeding (2 previous breast surgeries, 1 current treatment with drugs not compatible with breastfeeding), 1 for personal choice not to breastfeed, 10 for testing positive at nasopharyngeal swab for SARS-CoV-2, 11 for underlying maternal (1 cardiac disease, 1 psychiatric disorder) or neonatal (5 congenital infections, 3 respiratory distress, 1 urogenital malformation) conditions, 5 for non-availability of nasopharyngeal swab result at enrollment. Maternal and neonatal sociodemographics of the excluded population did not significantly differ from those of the enrolled population (*p* > 0.05). Eight mothers did not complete the questionnaire.

Most mothers (95%) chose the paper-based modality. The total number of questionnaires analyzed for the present study was 109. The response rate was 93%. Coincidentally, all of the enrolled mothers had a singleton pregnancy. The basic sociodemographic characteristics of the study population are summarized in Table 1. Participants’ characteristics were similar between the “SSN rooms” and “private rooms” mothers.

The exclusive breastfeeding rate at discharge was 80% and did not differ significantly between groups. The complementary feeding rate at discharge was 18%. Mothers switched to exclusive formula feeding at discharge in 2% of cases.

Table 2 summarizes the NPST scores of the enrolled mothers and the comparisons between the “SSN rooms” and “private rooms” mothers. Median NPST total score and median subsection scores did not differ among groups except for caregiving support, which resulted significantly higher in the “private rooms” group.

Mean TRAIT-A and STATE-A scores of the enrolled mothers are reported in Table 3: TRAIT-A and STATE-A scores were ≥40 in 30 % and 42% of mothers, respectively. Mean TRAIT-A and STATE-A scores did not significantly differ between the “SSN rooms” and “Private rooms” mothers. However, the percentage of “SSN rooms” mothers with a STATE-A score ≥of 40 was significantly higher than that of the “Private rooms” ones.

A TRAIT-A score ≥of 40 was significantly associated with a STATE-A score ≥of 40 at univariate binary regression, as did a NPST ≤ 4.23, being an “SSN mother” and being primiparous. Conversely, marital status, mode of delivery and maternal education did not correlate with a STATE-A score ≥ 40 at univariate analysis (*p* > 0.05). At multiple binary regression analysis, a TRAIT-A score ≥ 40, a NPST ≤ 4.23, being an “SSN mother”, and primiparity remained independently associated with a STATE-A score ≥ 40 (Table 4).

Among the variables of interest, only vaginal delivery and a priori choice to exclusively breastfeed were significantly associated with exclusive breastfeeding at discharge, both at univariate and multivariate binary logistic regression (Table 5). 

The mean anticipated duration of breastfeeding was 8.9 ± 4.6 months.

## 4. Discussion

The present study results indicate that non-infected mothers who delivered during the COVID-19 pandemic experienced high anxiety levels (STATE-A scores ≥40) in almost one out of two cases. Remarkably, the median STATE-A score of the mothers enrolled in the present study was higher than that of 162 mothers who delivered at the same hospital before the COVID-19 pandemic (median [IQR] STATE-A score 34 (28–43) vs. 36 (28.5–45.5), *p* = 0.023, respectively), among which the 30% showed STATE-A scores ≥40 vs. 42% of the enrolled mothers (*p* = 0.04) (authors’ unpublished data). Moreover, when investigating the differences between SSN rooms and private rooms, the partner’s presence may be considered beneficial. Mothers who could benefit from the constant presence of the newborn’s partner during hospital stay had STATE-A scores ≥40 in a significantly lower percentage of cases and a better perception of staff caregiving support which, in turn, may be related to a lower level of anxiety itself. Accordingly, although the maternal perception of staff support in our study was globally high, a higher NPST score was independently associated with a decreased risk of having a STATE-A score ≥ 40.

However, it must be considered that the more comfortable environment of the private rooms compared to the SSN rooms (single rooms vs. double or triple rooms) could also have contributed to the better perception of staff caregiving support reported by the mothers admitted to private rooms.

Among the reasons given to explain the decision not to breastfeed, one mother declared: “I would like to breastfeed, but because of the coronavirus, I do not know if I will receive enough specialized support and therefore if I will be able to manage everything”. Other common answers included nipple/breast pain, emotional distress, and work-related issues. As for breastfeeding rates at discharge, our results show that maternal anxiety levels and partner’s restricted policies were not associated with exclusive breastfeeding rates at discharge.

Of note, in our study population, breastfeeding rates at discharge were in line with those reported by previous studies performed in the same clinical setting in recent years [21]. This positive aspect could reflect a breastfeeding-supporting environment, as suggested by NPST median scores.

Maternal psycho-emotional vulnerability during catastrophic events is already well known [22]. Healthcare providers worldwide have been advocating a “call to action” to limit the impact of the restrictions imposed by the COVID-19 pandemic [23,24] on women’s perinatal medical care, focusing on psychological aspects in particular. Preliminary studies on pregnant and breastfeeding women during the COVID-19 pandemic in Belgium highlighted the need for targeted support, reporting a worsening of the Edinburgh Postpartum Depression Scale scores [25]. Moreover, a recent study in Japan compared Mother-to-Infant Bonding Scale scores before and during the COVID-19 pandemic, reporting a worsening in mother–infant bonding at one month postpartum during the pandemic [26]. Besides anxiety symptoms caused by the collectively-shared concerns about COVID-19, also described in the general population [27], newly mothers have faced the disruptive effect of lockdown and social distancing during pregnancy, labour, and delivery. In particular, during the hospital stay, the recommended isolation has inevitably posed an obstacle to staff one-on-one support and interaction between mothers admitted to the same ward, not to mention the forced deprivation of partners’ supportive role during the first days of life of the newborn.

Based on the present findings, allowing partners’ presence whenever possible could preserve maternal mental health and improve perceived hospital support. This could be particularly significant for primiparas, who were at higher risk of exhibiting a STATE-A score ≥ 40 compared to multiparas. Our results show that, besides cesarean section, a well-known barrier to breastfeeding [28], mothers’ a priori choice to breastfeed exclusively had a significant impact on breastfeeding rates at discharge. This result emphasizes the need to improve maternal education on breastfeeding importance during pregnancy and find new ways to achieve this goal in countries that are still experiencing lockdown and social distancing.

The absence of a follow-up is undoubtedly this study’s main limitation. Moreover, we cannot exclude that other confounding variables not considered in the present study might have affected our results; for example, other added stressors in SSN rooms, compared to private ones, such as the increased noise due to the presence of other dyads. Although in our study we could not demonstrate any significant association between exclusive breastfeeding at discharge and either maternal anxiety levels or partner’s presence/absence, it cannot be excluded that having experienced high anxiety levels or having been separated from the newborn’s partner after delivery could exert a negative long-term impact on breastfeeding rates and mental health outcomes. Accordingly, postnatal anxiety or depression is a well-known risk factor for breastfeeding difficulties and early breastfeeding cessation (30), both from a psychological and endocrinological point of view, due to the negative effect on lactation hormones as oxytocin [29,30,31].

## 5. Conclusions

The present study highlights the short-term effects of partner’s restricted visiting policies on non-infected newly mothers, underlining the importance of enabling partners to assist mothers during the hospital stay and supporting mothers, especially primiparas.

Breastfeeding rates at discharge were not influenced by maternal anxiety levels and restricted partner policies. Instead, they were influenced by mode of delivery, a well-known risk factor, and pre-pandemic intention to breastfeed, highlighting the importance of a mother’s own beliefs about breastfeeding.

As we all look forward to the decline of the COVID-19 pandemic, further studies are needed to explore its long-term effects on mothers’ mental health and breastfeeding rates.

## Figures and Tables

**Table 1:** Participants’ basic socio-demographic characteristics.

Socio-Demographic Characteristics	Mothers (*n* = 109)	SSN Rooms (*n* = 63)	Private Rooms (*n* = 46)	*p*-Value *
Age, median (IQR), y	35 (32–38)	35 (32–38)	35.5 (32–39)	0.38
Marital status, *n* (%)				0.75
Stable relationship	106 (97)	61 (97)	45 (98)	
Single	3 (3)	2 (3)	1 (2)	
Level of education, *n* (%)				0.35
≤13 years	41 (38)	26 (41)	15 (33)	
>13 years	68 (62)	37 (59)	31 (67)	
Pregnancy, *n* (%)				
Antenatal class	64 (59)	37 (59)	27 (59)	0.99
Primipara	70 (64)	36 (57)	34 (74)	0.07
Delivery, *n* (%)				
Cesarean section	38 (35)	24 (38)	14 (30)	0.40
Newborn				
Gestational age, median (IQR), weeks	39 (38–40)	39 (38–40)	39 (38–40)	0.75
Birthweight, mean (SD), g	3262.7 (429)	3298 (434)	3212.5 (422)	0.31
Mode of Feeding, *n* (%)				
Pre-pandemic intention to exclusively breastfeed	97 (89)	57 (90)	40 (87)	0.56
Exclusive breastfeeding at discharge	87 (80)	54 (86)	33 (72)	0.07

Abbreviations: SD, standard deviation; IQR, interquartile range. SSN, Servizio Sanitario Nazionale- Italian Public Healthcare System * SSN vs. private rooms.

**Table 2:** Nurse Parent Support Tool (NPST) results.

NPST Results	Mothers (*n* = 109)	SSN Rooms (*n* = 63)	Private Rooms (*n* = 46)	*p*-Value *
MEDIAN SCORE	4.23 (3.3–4.6)	4.04 (3.2–4.6)	4.38 (3.5–4.7)	0.75
Informational Support	4.11 (3.2–4.5)	4.00 (3.2–4.5)	4.22 (3.3–4.6)	0.15
Emotional Support	4.00 (3.0–4.6)	4.00 (2.6–4.6)	4.00 (3.3–4.6)	0.15
Appraisal/Parental Esteem Support	4.25 (3.2–4.7)	4.00 (3.0–4.7)	4.25 (3.2–4.7)	0.75
Caregiving Support	4.60 (4.0–5.0)	4.40 (3.6–5.0)	4.80 (4.1–5.0)	0.04

NPST results expressed as median (IQ); Abbreviations: IQR, interquartile range, SSN, Servizio Sanitario Nazionale- Italian Public Healthcare System * SSN vs private rooms.

**Table 3:** State-Trait Anxiety Inventory-Form Y (STAI-Y) results.

STAI-Y Results	Mothers (*n* = 109)	SSN Rooms (*n* = 63)	Private Rooms (*n* = 46)	*p*-Value
TRAIT-A score, mean (SD)	34.6 (8.0)	35.2 (7.5)	33.7 (8.7),	0.2
STATE-A score, mean (SD)	38 (11.9)	39.1 (12.6)	36.4 (10.8)	0.3
TRAIT-A score ≥ 40, *n* (%)	33 (30)	20 (312)	13 (28)	0.7
STATE-A score ≥ 40, *n* (%)	46 (42)	32 (51)	14 (30)	0.03

Abbreviations: SSN, Servizio Sanitario Nazionale—Italian Public Healthcare System; SD, standard deviation; TRAIT-A, personality trait; STATE-A, concurrent emotional state.

**Table 4:** Binary logistic regression analyses predicting the likelihood of having a STATE-A ≥40.

Variable	Univariate Analysis			
	OR	95% CI for OR		*p*- Value
		**LL**	**UL**	
TRAIT-A score (≥40 vs. <40)	2.95	1.27	6.88	0.012
Having a NPST (≤4.23 vs. >4.23)	5.66	2.44	13.14	<0.0005
“SSN room” vs. “private room”	2.36	1.06	5.25	0.035
Maternal age (≤35 vs. >35 y)	1.93	0.88	4.23	0.098
Marital status (single vs. engaged in a stable relationship)	0.35	0.03	4.03	0.40
Maternal education (>13 vs. ≤13 y)	1.05	0.47	2.30	0.90
Parity (primipara vs. multipara)	2.54	1.09	5.89	0.029
Mode of delivery (cesarean section vs. vaginal delivery)	1.63	0.73	3.61	0.229
	**Multivariate Analysis**			
	**OR**	**95% CI for OR**		***p*-Value**
TRAIT-A score (≥40 vs. <40)	3.45	1.27	9.35	0.015
Having a NPST (≤4.23 vs. >4.23)	4.72	1.91	11.614	0.001
“SSN room” vs. “private room”	2.73	1.06	7.07	0.037
Parity (primipara vs. multipara)	3.74	1.35	10.37	0.011

Abbreviations: NPST, Nurse Parent Support Tool; SSN, Servizio Sanitario Nazionale—Italian Public Healthcare System; OR, Odds Ratio; CI, Confidence Interval; LL, Lower Limit; UL, Upper Limit.

**Table 5:** Binary logistic regression analyses predicting the likelihood of exclusive breastfeeding at discharge.

Variable	Univariate Analysis			
	OR	95% CI for OR		*p*-Value
		**LL**	**UL**	
STATE-A score (≥40 vs. <40)	0.67	0.26	1.72	0.40
TRAIT-A score (≥40 vs. <40)	1.2	0.42	3.40	0.73
Having a NPST (≤4.23 vs. >4.23)	0.64	0.25	1.66	0.36
“SSN room” vs. “private room”	2.36	0.91	6.13	0.077
Maternal age (≤35 vs. >35 y)	1.7	0.66	4.35	0.27
Marital status (single vs engaged in a stable relationship)	2.02	0.17	23.39	0.57
Maternal education ( >13 y vs. ≤13)	1.19	0.46	3.09	0.72
Parity (primipara vs multipara)	0.45	0.15	1.35	0.16
Mode of delivery (vaginal delivery vs. cesarean section)	2.81	1.08	7.32	0.034
Antenatal class (yes vs. no)	1.23	0.48	3.17	0.65
Pre-pandemic intention to exclusively breastfeed (yes vs. no)	7.65	2.14	27.32	0.002
	**Multivariate Analysis**			
	**OR**	**95% CI for OR**		***p*-Value**
Mode of delivery (vaginal delivery vs. cesarean section)	3.35	1.18	9.52	0.023
Pre-pandemic intention to exclusively breastfeed (yes vs. no)	9.12	2.37	35.1	0.001

Abbreviations: NPST, Nurse Parent Support Tool; SSN, Servizio Sanitario Nazionale—Italian Public Healthcare System; OR, Odds Ratio; CI, Confidence Interval; LL, Lower Limit; UL, Upper Limit.

## Data Availability

The data presented in this study are available on request from the corresponding author. The data are not publicly available due to privacy restrictions.
